# Road traffic injured patients with severe GCS and organ injury had a poor prognosis: a retrospective cohort study

**DOI:** 10.1186/s12889-019-7100-y

**Published:** 2019-06-13

**Authors:** Kissanet Tesfay, Mulubirhan Assefa, Dawit Zenebe, Mekonnen Gebremicael, Getahun Kebede, Hayelom Gebrekirstos

**Affiliations:** 1 Department of Epidemiology, Mekelle University College of Health Science School of Public Health, Mekelle, Ethiopia; 2 Tigray Regional Health Bureau, Mekelle, Ethiopia; 3 Adigrat University College of Health Science Department of Pharmacy, University of Saskatchewan, School of Public Health, Mekelle, Ethiopia

**Keywords:** Road traffic injury, Glass coma scale, Ayder comprehensive specialized referral hospital

## Abstract

**Background:**

Ethiopia had an increasing trend of morbidity and mortality due to road traffic injury. Road traffic injured patient’s recovery rate is affected by many different factors. Those factors might affect the duration of time to recovery. Therefore studying the median time to recovery and its predictors of road traffic injured patients will be needed to act upon the patient’s hospital provided service.

**Method:**

A retrospective cohort study design was employed. The study population was all admitted road traffic injured patients in Ayder tertiary hospital. We have used the total of all three-year RTI patients’ chart from 2015 to 2017 found in the hospital. After excluding incomplete charts for major variables the sample size was 322. Descriptive statistics, life table, Kaplan-Meier, log-rank test and assumptions of the Cox proportional hazard model was applied. Bi and multivariate Cox regression analysis, hazard ratios and associated 95% CI were estimated.

**Result:**

Male to female RTI patient ratio was 3:1. Of the total 258(80.1%) had been recovered and the median survival time to recovery was 15 days (interquartile range 7–29). From those recovered, 104(40.3%) had been referred from other health facilities. Availability of referral form linkage [adjusted hazard ratio = 1.5, CI (1.1–1.9)], mild and moderate glass coma scale [adjusted hazard ratio = 2.3, CI (1.3–3.9)], conservative management [adjusted hazard ratio = 1.6, CI (1.2–2.1)], and not having organ injury [adjusted hazard ratio = 1.6, CI (1.1–2.3)] were associated with time to recovery in multivariate analysis.

**Conclusion:**

Median time to recovery of road traffic injured patients was relatively good. Being referred from another health facility, mild and moderate glass coma scale, conservative management and without organ injury was positively associated with time to recovery of road traffic injured patients. We would like to recommend for future prospective studies to determine the time to return to work of road traffic injured patients and quality of life after the injury.

## Background

One of the sustainable development goal (SDG) targets was to reduce 50% of global road traffic collision deaths and injuries by 2020 [1]. However different studies conducted in Ethiopia showed that the trend of morbidity and mortality of road traffic injury (RTI) in the country had been increasing from year to year [2, 3]. According to, 2013 WHO’s Global Status Report on Road Safety, Ethiopia had 68.3 deaths of RTI per 10,000 vehicles.

People with low socioeconomic status, males and young age groups were at high risk of road traffic injury [4]. Road traffic injured patient’s recovery rate is affected by many different factors. Different studies have identified factors such as socio-demographic factors, crash characteristics, type of pre-hospital care given, hospital arrival mechanism, injury characteristics, and hospitalization care. Among those factors sex, age, glass coma scale (GCS) score, injury severity score (ISS) and systolic blood pressures at admission were increasingly associated with recovery from road traffic injured patients [5–10].

However, in Ethiopia as well in Ayder Comprehensive Specialized Referral Hospital little is known about epidemiological studies conducted on time to recovery and its predictor factors of road traffic injured patients. Therefore it is important that survival study is conducted to determine the time to recovery and its predictor factors among admitted road traffic injured patients.

## Methods

### Study area and period

The study was conducted in Ayder Comprehensive Specialized Referral Hospital from 2015 to 2017. Data was collected from March 1 to 30, 2018. The hospital is found in northern Ethiopia located 778 km from Addis Ababa. The hospital had 513 inpatient beds with three intensive care units.

### Study design

Hospital-based retrospective cohort study design on RTI patient’s record was conducted.

### Population

#### Source population

All admitted road traffic injured patients in Ayder Comprehensive Specialized Referral Hospital from 1 January 2015 to 30 December 2017.

#### Study population

All road traffic injured patients from 1 January 2015 to 30 December 2017 admitted at Ayder Comprehensive Specialized Referral Hospital.

#### Study unit

All road traffic injured patients chart in Ayder Comprehensive Specialized Referral Hospital from 1 January 2015 to 30 December 2017.

### Eligibility criteria

All medical records of road traffic injured patients who were admitted to Ayder Comprehensive Specialized Referral Hospital from 2015 to 2017 were included. Whereas, incomplete patient chart (for major variables) and repeat patients on RTI during the study time were excluded.

### Sample size determination

The sample size was calculated using the stpower Cox STATA software version 12. The calculation was done based on the assumption that type I error of 5%, power of 80% and a standard deviation of 0.5. From a previous study conducted in Turkey, the overall probability of recovery from a traumatic brain injury was 50% and hazard ratio for glass coma scale (survivals vs. non-survivals) was 0.643 [11]. Glass coma scale is a most common predictor in most literature. The total sample size was 322.

### Sampling procedure

All three-year records of RTI patients chart found in Ayder Comprehensive Specialized Referral Hospital were included. The total of three-year RTI patients chart was 342. We excluded 20 charts due to incompleteness for major variables such as length of stay, sex, age, GCS.

### Data collection tool and procedures

English version data extraction check-list was developed and adapted from different works of literature. Data were extracted from registers, patient’s card, intensive care unit (ICU) and operation notes using a data extraction tool for the occurrence of the event. The event was recovery from RTI.

The checklist consists variables such as socio-demographic variables, availability of referral linkage, patient blood pressure at admission, information regarding the injury, previous history of medical illness, the location of the injury, type of injury (Injury severity score), length of hospital stays, ICU admission, organ injury and patient management. The data was collected by three nurses that had a Bachelor of Science degree.

### Study variables

#### Dependent

Time to recovery from road traffic injury.

#### Independent

Socio-demographic factors include age, sex, and address.

Patients’ factors include the availability of referral form linkage, duration of RTI, the situation of patients during RTA and previous history of medical illness.

Clinical factors of patients include consciousness status at admission, blood Pressure at admission, the location of the injury, GCS, KTS II, patient management, organ injury, ICU admission and length of hospital stays.

### Data quality control

Three days training was given to data collectors on the data collection checklist and technique. A pre-test was undertaken on the checklist in 5% at Ayder Comprehensive Specialized Referral Hospital before the actual data collection started. Amendment was made on the checklist for clarity and consistency. Every day during the data collection process the checklist was checked for completeness, consistency, and accuracy by the principal investigator.

### Data processing and analysis

The data was coded; entered, and cleaned using SPSS version 21. And assumptions were checked using STATA version 12. The levels of missing values, the presence of influential outliers, multicollinearity, normality, and proportionality of hazards over time have been checked. Proportionality of hazard over time was met and the mean-variance inflation factor was 1.86.

Descriptive statistics, life table, Kaplan-Meier (KM) survival, and hazard functions were applied to estimate the probability of recovered patients from RTI and Log-rank test was used to compare the KM curves for two or more categories of RTI patients.

The model for Cox proportional hazard is [12]

$$ h\left(t,X\right)=h0(t)\mathit{\exp}\left[\sum \upbeta ixi\right] $$

Where;

hx(t) = hazard function

h0(t) = baseline hazard

exp.(βiXi) = function reflects how the hazard function changes according to difference in subjects’ characteristics (X)

Cox proportional hazard model was used to determine the relationship between independent variables and the outcome variable (time to recovery from RTI). First bivariate Cox-regression analysis was done to estimate the crude hazard ratios (CHR). Every independent variable was tested against the dependent variable and variables significant at *P* < 0.25 in bivariate analysis and those which were significant predictors in the most studies was taken to the multivariate Cox regression model. Then multivariate Cox-regression analysis was performed to estimate the Adjusted Hazard Ratios (AHR). AHR with 95% Confidence Interval (CI) was used to measure the association between dependent and independent variables.

### Operational definitions

Event (1) was defined as the occurrence of recovery from road traffic injury.

Time to recovery was defined as time to occurrence of recovery from a road traffic accident.

Recovery was defined as when RTI patients’ chart showed that the patient had improved with or without permanent disability and discharged.

Censored (0) was when a patient is transferred, discharge against medical advice, study time completion and died.

Glass coma scale was classified as severe (GCS 3–8), moderate (GCS 9–12) and mild (GCS 13–15) [5].

Kampala trauma severity Score II (KTS II) was classified as mild injury 9–10, moderate injury 7–8 and severe injury 6 or less (≤6) [13].

Permanent disability includes light damage with minor neurological and psychological deficits or no need for assistance in everyday life; employment is possible but may require special equipment or permanent need for help with daily living [9].

Availability of referral form is defined as when a patient comes with a written referral form from referring health facility attached with a patient chart or if there is a written history of being referred from another health facility.

## Result

### Socio-demographic factors

The overall recovery rate was 258(80.1%), and 64(19.9%) was censored. From the 258 recovered road traffic injured patients 134(51.9%) of them had a permanent disability.

The mean and standard deviation of RTI patients’ age was 30.1 ± 15.9. Male to female ratio of RTI victims was 3:1. Socio-demographic characteristics overall and stratified by recovery status are presented in Table 1.

**Table 1:** Socio-demographic characteristics of RTI victims admitted at ACSRH, Mekelle, Ethiopia, 2015–2018 (*N* = 322)

Socio-demographic characteristics	Recovered(*N* = 258)	Censored (*N* = 64)	Total (*N* = 322)
Age of patients			
0–14	38(14.7%)	5(7.8%)	43(13.3%)
15–24	64(24.8%)	10(15.6%)	74(23%)
25–35	84(32.6%)	16(25%)	100(31.1%)
> 36	72(27.9%)	33(51.6%)	105(32.6%)
Sex of patients			
Male	197(76.4%)	49(76.6%)	246(76.4%)
Female	61(23.6%)	15(23.4%)	76(23.6%)
Residence of patients			
Urban	111(43.0%)	29(45.3%)	140(43.5%)
Rural	147(57%)	35(54.7%)	182(56.5%)
Ethnicity			
Tigrawi/ti	234(90.7%)	59(92.2%)	293(91.0%)
Other^a^	24(9.3%)	5(7.8%)	29(9%)

Other^a^ include Afar, Amhara, and Benshangul gumuz

### Patient factors

RTI patients with referral form were 104(40.3%). Recovered patients arrived at the hospital within 12 h of the occurrence of the injury was 71.7%. Passengers accounted for 144(44.7%) (Table 2).

**Table 2:** Characteristics of the road traffic injured patients admitted to ACSRH: 2015–2017 (*N* = 322)

Patient factors	Recovered(*N* = 258)	Censored (*N* = 64)	Total (*N* = 322)
Availability of referral form linkage			
Yes	104(40.3%)	15(23.4%)	119(37%)
No	154(59.7%)	49(76.6%)	203(63%)
Duration after RTC collusion			
< 12 Hours	185(71.7%)	51(79.7%)	236(73.3%)
12–24 Hours	26(10.1%)	2(3.1%)	28(8.7%)
> 24 Hours	47(18.2%)	11(17.2%)	58(18%)
The situation of patients during RTA			
Passengers	119(46.1%)	25(39.1%)	144(44.7%)
Pedestrian	90(34.9%)	32(50.0%)	122(37.9%)
Driver	46(17.8%)	7(10.9%)	53(16.5%)
Other	3(1.2%)	0	3(0.9%)
History of previous medical illness			
Yes	11(4.3%)	7(10.9%)	18(5.6%)
No	247(95.7%)	57(89.1%)	304(94.4%)

### Clinical factors

RTI patients admitted into surgical ward 205(63.7%), Orthopedics surgery ward 60(18.6%), Pediatrics ward 45(14%), Neurosurgery ward 6(1.9%) and Gynecology ward 1(0.3%).

Among the admitted RTIs 160(49.7%) were conscious of admission. Extremity injury of admitted RTI patients accounted for 171(53.1%) (Table 3).

**Table 3:** Clinical factors of road traffic injured patients admitted at ACSRH, 2015–2017 (*N* = 322)

Variables	Recovered (*N* = 258)	Censored(*N* = 64)	Total(*N* = 322)
History of loss of consciousness at admission			
Conscious	137(53.1%)	23(35.9%)	160(49.7%)
Unconscious	121(46.9%)	41(64.1%)	162(50.3%)
Admission blood pressure			
< 90/60	9(3.5%)	10(15.6%)	19(5.9%)
90/60–140/90	246(95.3%)	51(79.7%)	297(92.2%)
> 140/90	3(1.2%)	3(4.7%)	6(1.9%)
Injured body part			
Extremities injury	156(60.5%)	15(23.4%)	171(53.1%)
Head injury	110(42.6%)	47(73.4%)	157(48.8%)
Chest injury	25(9.7%)	8(12.5%)	33(10.2%)
Back injury	10(3.8%)	6(9.4%)	16(4.9%)
Other	18(6.9%)	5(7.8%)	23(7.1%)
Multiple Injury			
Yes	61(23.6%)	17(26.6%)	78(24.2%)
No	197(76.4%)	47(73.4%)	244(75.8%)
GCS at admission			
Mild (13–15) and moderate (9–12)	93(36.1%)	17(26.6%)	110(34.2%)
Sever(<=8)	17(6.5%)	30(46.8%)	47(14.6%)
NA	148(57.4%)	17(26.6%)	165(51.2%)
KTS II			
Moderate (7, 8)	238(92.2%)	26(40.6%)	264(82%)
Severe(≤6)	20(7.8%)	38(59.4%)	58(18%)
Type of patient management			
Surgical	155(60.1%)	27(42.2%)	182(56.5%)
Conservative	103(39.9%)	37(57.8%)	140(43.5%)
Admission to ICU			
No	227(88%)	29(45.3%)	256(79.5%)
Yes	31(12%)	35(54.7%)	66(20.5%)
Organ Injury			
No	226(87.6%)	15(23.4%)	241(74.8%)
Yes	32(12.4%)	49(76.6%)	81(25.2%)
Length of Hospital Stay (LOS)			
< 14 Days	136(52.7%)	53(82.8%)	189(58.7%)
15 to 24 Days	43(16.7%)	3(4.7%)	46(14.3%)
25 to 34 Days	33(12.8%)	5(7.8%)	38(11.8%)
> 35 Days	46(17.8%)	3(4.7%)	49(15.2%)

### The median time to recovery of admitted RTI patients

A total of 322 patients were followed for three different year period which produced a total of 5387 person day observation. The incidence of recovery was 5 per 100 person-day observation.

The cumulative survival probability of admitted RTI patients was 90.8% (95%CI, 86.9–93.5) at day three, 79.8% (95%CI, 74.7–83.9) at day six, 50% (95% CI, 44.1–56) at day fifteen and 41.5% (95%CI, 35.5–47.4) at day twenty-one. The overall cumulative survival probability was 0.5% (95% CI, 0.05–2.5).

**Fig. 1:**
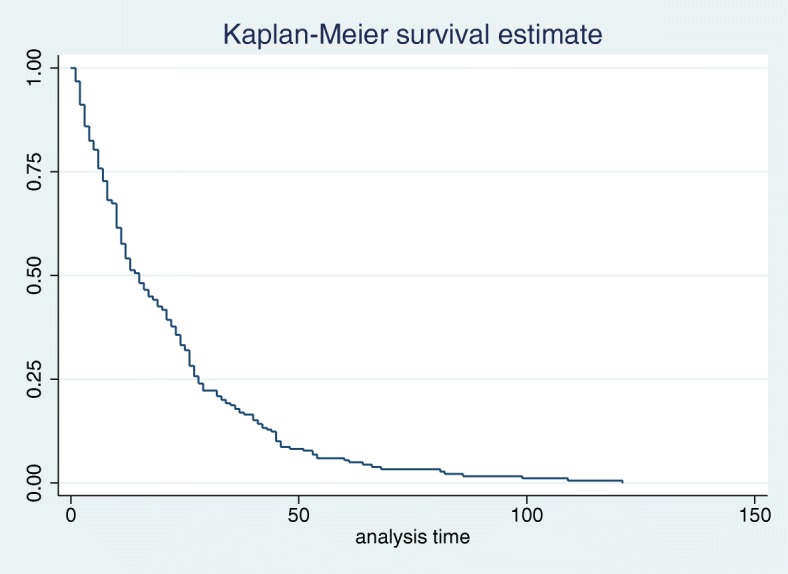
Kaplan–Meier curve of time to recovery of RTI patients in ACSRH, 2015–2017

The median time to recovery of admitted road traffic injured patients was 15 days (interquartile range (IQR) 7, 29) Fig. 1.

The log-rank test showed that there was a statistically significant difference in time to recovery of availability of referral form, ICU admission, admission GCS, organ injury, KTS II and patient management (Table 4).

**Table 4:** Log-rank test of time to recovery among covariates in ACSRH 2015–2017 (*N* = 322)

Variables	Chi-square	Degree of freedom	*P*-value
Age	2.93	3	0.40
Sex	1.79	1	0.18
Place of residence	1.72	1	0.19
Availability of referral form	7.96	1	0.005^*^
Duration before hospital arrival	0.34	2	0.84
The situation of patients during RTA	0.664	3	0.88
Comorbidity	3.16	1	0.08
Consciousness status	0.33	1	0.56
Admission BP	1.28	2	0.53
Multiple injury	0.96	1	0.33
Admission GCS	13.2	1	0.000^*^
KTS II	4.48	1	0.03^*^
Management	13.14	1	0.000^*^
ICU admission	5.6	1	0.018^*^
Organ injury	6.2	1	0.012^*^

^*^for the *p*-value of< 0.05

### Assessment of model adequacy

**Fig. 2:**
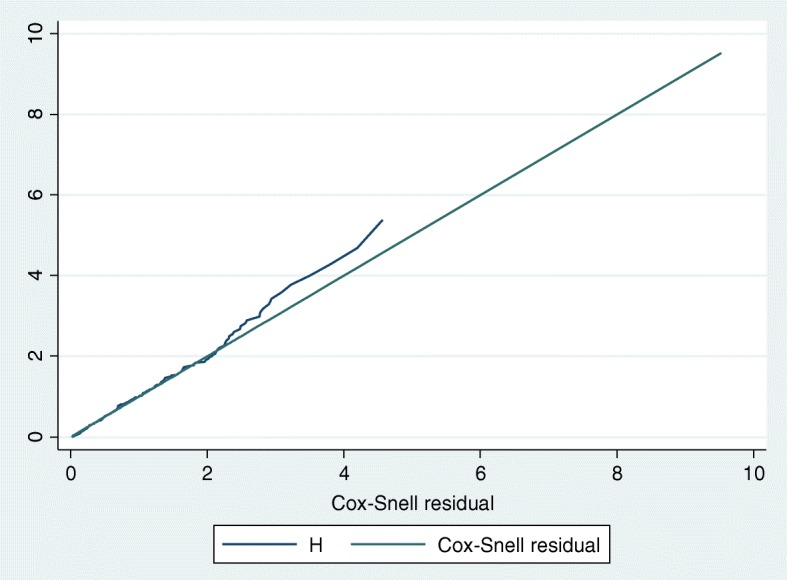
Cox-Snell residual plot for checking the assumption of the model, on RTIs in ACSRH, 2015–2017

The proportional hazards model assumption is one very important assumption in the Cox model. The graphical and statistical method was used to assess the assumption. The Cox Proportional Hazard model assumption was checked by Cox-Snell residual plot. The plot showed that the hazard follows the 45-degree line very closely and we conclude that the data was fitted well (Fig. 2).

Additionally, the Cox proportional hazard model assumption was checked by using Scaled Schoenfeld residual test (global test *P* = 0.76) which indicated that the assumption was met.

### Predictors of time to recovery

In Multivariate Cox PH model; availability of referral form 1.5(1.1–1.9), admission GCS 2.3(1.3–3.9), organ injury 1.6(1.1–2.3), and patient management 1.6(1.2–2.1) was significantly associated with recovery (Table 5).

**Table 5:** Cox Proportional Hazards Regression for time to recovery of RTIs in ACSRH, 2015–2017(*N* = 322)

Predictors	Crude hazard ratio (95%CI)	Adjusted hazard ratio (95%CI)
Sex		
Female	1	–
Male	0.82(0.61–1.10)^*^	–
Residence		
Urban	1	–
Rural	0.835(0.65–1.07)^*^	–
Availability of referral form		
No	1	1
Yes	1.43(1.06–1.84)^*^	1.5(1.13–1.91)
History of previous medical illness		
No	1	–
Yes	0.59(0.32–1.08)^*^	–
KTS II		
Sever	1	–
Moderate	1.63(1.02–2.61)^*^	–
Admission GCS		
Severe	1	1
Mild and moderate	2.53(1.48–4.31)^*^	2.26(1.31–3.88)
ICU admission		
No	1	–
Yes	0.64(0.44–0.94)^*^	–
Patient management		
Surgical	1	1
Conservative	1.59(1.2–2.06)^*^	1.6(1.19–2.13)
Organ injury		
Yes	1	1
No	1.58(1.09–2.29)^*^	1.6(1.09–2.31)

^*^variables with *p* < 0.25

## Discussion

Our study result showed that median time to recovery was 15 days (IQR, 7–29) with 80.1% of overall recovery rate. Time to recovery was positively associated with having referral form, conservative management, not having organ injury, and mild and moderate admission GCS.

This study finding showed that the median time to recovery of RTI patients was 15 days (IQR, 7–29). This was in line with the median recovery time of studies done in Malawi (16 days), and South Africa (10 days) [14, 15]. However, our study finding differs from a study conducted in Kenya (30 days) [16]. The discrepancy might be due to that in Kenya there was a high number of extremity injured patients (56.4%), while in this study it was 49.7% (95% CI, 45–55.6%). This could be due to the reason that extremity injury takes a long time to recover [17].

Though there is no standard to what constitutes adequate time to recovery for RTI patients our study identified a relatively good time to recovery compared with other studies conducted across Africa. But in our study, about 47 % of severe GCS and 77 % of organ injured RTI patients hadn’t recovered which needs special attention.

In this study, the overall recovery rate of RTI patients was 80.1% (95% CI, 75.2–84.5%). This was in agreement with a cross-sectional study conducted in Tikur Anbesa of Ethiopia that had 77.5% of recovery rate [8]. And a retrospective cohort study of Tehran, Iran with 79.2% of RTI patient’s recovery rate [9]. However our study finding was lower than cross-sectional studies done in Wolayta zone and Dire Dawa of Ethiopia, and Tanzania indicated that RTI patient’s recovery rate was more than 90% [5, 10, 18]. This disparity could be due to that in our study we included only admitted RTI patients whereas, in the studies done in Wolayta of Ethiopia, and Tanzania they had included all inpatient and outpatient trauma victims visited their hospital.

This study revealed that most of the recovered RTI patients 71.7% (95% CI, 67.2–76.4) presented to the hospital within 12 h of the injury occurrence. This finding was higher than the study finding of Dire Dawa, Ethiopia which was 59.2% [10]. The difference could be due to the reason that Dire Dawa’s RTI patient’s arrival with the referral was 11% that was lower than compared to our study finding 37% (95% CI, 31.7–42.2). This might indicate for late patient hospital arrival in Dire Dawa study.

In this study, 52.7% (95% CI, 47.7–58) of recovered RTI patients had less than 2 weeks of a length of hospital stay (LOS). This disagrees with study result of Dire Dawa, Ethiopia that had 19.4% of recovered RTI patients that had less than 2 weeks of LOS [10]. This might be due to the difference in time of presence of RTI patients to the health facility.

This study revealed that RTI patients with available referral form were recovered more than those who came without referral form. This was in line with a study done in India [19]. This might be due to the reason that if patients come with referral paper, this could indicate they have got immediate access to first aid service and resuscitation in the nearby health facility. This could also indicate that referred patients might get special service regarding their referral reason.

The finding of our study showed that RTI patients with mild and moderate GCS score were more likely to recover than those with severe GCS score. This was in agreement with studies conducted in Kenya and Turkey [11, 16]. This might be due to the reason that traumatic brain injured RTI patients who sustain a mild or moderate GCS have increased consciousness level and coordination than that of with severe GCS score. GCS is a quantifiable determination used for traumatic brain injured patients prognosis [17].

Our study finding of KTS II was not associated with RTI patient’s recovery. However, this was not in line with the study result of Tanzania that showed severe KTS II was associated with unrecovered patients [5]. And also studies conducted in Kenya and Tehran of Iran showed increased injury severity score was associated with unrecovered patients [9, 16]. The difference with study in Tanzania could be due to the Tanzania study was prospective, whereas our study was retrospective. And the difference with studies of Kenya and Tehran of Iran could be due to that, they have used injury severity score (ISS) classification while we have used the Kampala injury severity score II (KTS II) classification.

In the present study, patients managed by conservative management were more likely to recover than those with surgical management. This is in agreement with studies conducted in Turkey and Dire Dawa of Ethiopia [10, 11]. This might be due to conservative management had no risk of surgical site infections which might not lead to prolonged hospital stay and complications.

Our study finding showed that RTI patients without organ injury were more likely to recover than those with organ injury. This was in line with a study conducted in Tehran of Iran [9]. The possible explanation could be due to organ damage causes fatal internal and external bleedings and organ failure which might lead to poor recovery and death.

The strength of this study was in an attempt done to conduct a study in time to recovery and its predictor factors among admitted RTI patients.

The limitation of the study was the data was secondary so important variables may be missed. The study lacks literature about time to recovery of RTI patients. There was no standard point of reference for time to recovery. And also the status and quality of life of RTI patients after discharge from hospital were not known.

## Conclusion

Generally, the median time of recovery of admitted RTI patients was relatively high. Being referred from another health facility, conservative management, not having organ injury and mild and moderate admission GCS were found to be associated with better time to recovery of admitted RTI patients.

Ayder Comprehensive Specialized Referral Hospital should give attention to patients with the identified factors. Additionally, we would like to recommend for future prospective studies to determine the time to return to work of road traffic injured patients and quality of life after the injury.

## Data Availability

The datasets used and/or analyzed during the current study are available from the corresponding author on reasonable request.

## Abbreviations

ACSRH: Ayder Comprehensive Specialized Referral Hospital
AHR: Adjusted Hazard Rate
CHR: Crude Hazard Rate
CI: Confidence Interval
GCS: Glasgow Coma Scale
HR: Hazard Rate
ICU: Intensive Care Unit
IQR: Inter-Quartile Range
ISS: Injury Severity Score
KTS: Kampala Trauma Score
LOS: Length of Hospital Stay
RTI: Road Traffic Injuries
SBP: Systolic Blood Pressure
SDG: Sustainable Development Goal
TBI: Traumatic Brain Injury
WHO: World Health Organization
